# Supplementary material for: Food and fluid intake during labor in maternity wards: A scoping review protocol
Source: PLoS One. 2024 Sep 18;19(9):e0309746. doi: 10.1371/journal.pone.0309746 (PMC11410181; doi:10.1371/journal.pone.0309746)
Supplement: Attachment — Submitted filename: Plos_Reviewer Comments.docx [file pone.0309746.s003.docx]

Thank you for allowing me to review the scoping review protocol. The proposed scoping review, which addresses a crucial and timely topic, is of significant importance. I have carefully reviewed the changes made by the authors to the first round of review. These changes have notably enhanced the protocol, making it much clearer. However, there are a few minor adjustments that could further improve the protocol, as outlined below.

**Abstract**

Include a statement on how data extraction will be done.

**Introduction**

The authors state that this review will build on the work done by Signata et al. Please add the review dates covered by the review of Signata et al. in line 67.

Line 79: The Joanna Briggs Institute was renamed to JBI. Please use the updated name. The same comment applies to line 88

**Review question**

The authors refer to “usual-risk labour.” Define this concept in the introduction to make it clear to readers.

**Inclusion criteria**

Will the reviewers have any exclusion criteria? If yes, please include. If not, please state that there is no exclusion criteria.

**Source Types**

Line 116: The authors state that papers in all languages will be included. Please describe how studies in other languages will be searched for and how the findings will be reported.

Line 117: Seems to state that there were exclusion criteria. Please clarify. Also, clarify if the sentence in lines 117 and 118 should be in past tense.

**Search strategy**

Line 149: change the word “research” to “search.”

Line 152: Please clarify how these terms will be used to search papers in languages other than English.

**Study selection**

Line 173 states that there are exclusion criteria. Please state what it is.

Line 179: Please clarify: Will the references to included articles be analysed for inclusion in the review, or are the authors meant to say that references to included articles will be analysed to identify additional articles for inclusion?
