# Supplementary material for: Food and fluid intake during labor in maternity wards: A scoping review protocol
Source: PLoS One. 2024 Sep 18;19(9):e0309746. doi: 10.1371/journal.pone.0309746 (PMC11410181; doi:10.1371/journal.pone.0309746)
Supplement: Attachment — Submitted filename: Response to Reviewers.docx [file pone.0309746.s004.docx]

FEDERAL UNIVERSITY OF RIO GRANDE DO NORTE

GRADUATE PROGRAM IN HEALTH SCIENCE

Santa Cruz/RN, 31 de july de 2024.

Dear publisher,

We hereby submit the corrected and revised manuscript entitled **“Food and fluid intake during the labor process: a scoping review protocol”**, authored by Brenda Kelly Pontes Soares, Byanca Rodrigues Carneiro, Ilana Bruna de Lima Feitoza, Anna Cecilia Queiroz de Medeiros and Adriana Gomes Magalhães, for your consideration.

First of all, we appreciate the interest in our article and would like to thank the reviewers of this study, who provided very pertinent comments, contributing to the improvement of our manuscript. After analyzing the considerations and questions, the article has undergone some changes, which are indicated below.

Thanks in advance for your attention.

Brenda Kelly Pontes Soares.

**ANSWERS TO THE PUBLISHER’S QUESTIONS**

The Editor suggested the highlighted revisions below:

**QUESTION 1) Implications for practice and Recommendations**

***ANSWER:*** *In order to meet the reviewer’s request, the possible contributions/implications for clinical practice that may arise from the results of this scoping review were inserted in the ‘Discussion’ section on (lines 262-267).*

**QUESTION 2) Flow diagram for systematic protocol which included searches of databases, registers and other sources used.**

***ANSWER:*** *As stated in the protocol, the PRISMA flowchart will be used to present the results of the scoping review, which has been inserted in line 229-230. However, to address the reviewer's request, a figure summarizing the information about the structure of the scoping review has been developed (line 107).*

**RESPONSES TO THE REVIEWERS QUESTIONS:**

**RESPONSE TO REVIEWER #4.**

Reviewer #4 suggested the following rewordings:

**QUESTION 1) Abstract: Include a statement on how data extraction will be done.**

***ANSWER:*** *The correction has been made. A statement on how data extraction will be conducted has been included (lines 47-48).*

**QUESTION 2) Introduction: The authors state that this review will build on the work done by Signata et al. Please add the review dates covered by the review of Signata et al. in line 67.**

***ANSWER:*** *The review dates covered by the review of Signata et al. have been added (lines 77-78).*

**QUESTION 3) Introduction: Line 79: The Joanna Briggs Institute was renamed to JBI. Please use the updated name. The same comment applies to line 88.**

***ANSWER:*** *The updated name has been added in line 37, 90, 99 and 141 as per the reviewer's suggestions.*

**QUESTION 4) Review question:** **The authors refer to “usual-risk labour.” Define this concept in the introduction to make it clear to readers.**

***ANSWER:*** *The concept of "habitual risk work" has been defined in the introduction to provide greater clarity for the readers (lines 71-73).*

**QUESTION 5) Inclusion criteria:** **Will the reviewers have any exclusion criteria? If yes, please include. If not, please state that there is no exclusion criteria.**

***ANSWER:*** *Yes, we will have exclusion criteria. We have included information about the exclusion of studies in the "Inclusion and Exclusion Criteria" section (lines 112-125).*

**QUESTION 6) Source Types: Line 116: The authors state that papers in all languages will be included. Please describe how studies in other languages will be searched for and how the findings will be reported.**

***ANSWER:*** *No specific search strategy will be adopted for Portuguese and Spanish languages, as it is required to present a version of the abstract, title, and keywords in English in all the databases investigated.*

**QUESTION 7) Source Types:** **Line 117: Seems to state that there were exclusion criteria. Please clarify. Also, clarify if the sentence in lines 117 and 118 should be in past tense.**

***ANSWER:*** *Information about the inclusion criteria has been clearly included in the "Inclusion and Exclusion Criteria" section (lines 112-125). Additionally, the sentence in lines 117 and 118 has been revised, and the verb has been corrected to the future indicative tense (lines 194-195).*

**QUESTION 8) Search strategy: Line 149: change the word “research” to “search.”**

***ANSWER:*** *The writing has been revised according to the request from reviewer #4 (line 168).*

**QUESTION 9) Study selection:** **Line 152: Please clarify how these terms will be used to search papers in languages other than English.**

***ANSWER:*** *No specific search strategy will be adopted for Portuguese and Spanish languages, as it is required to present a version of the abstract, title, and keywords in English in all the databases investigated.*

**QUESTION 10) Study selection: Line 173 states that there are exclusion criteria. Please state what it is.**

***ANSWER:*** *Information about the inclusion criteria has been clearly included in the "Inclusion and Exclusion Criteria" section (lines lines 112-125).*

**QUESTION 11) Study selection: Line 179: Please clarify: Will the references to included articles be analysed for inclusion in the review, or are the authors meant to say that references to included articles will be analysed to identify additional articles for inclusion?**

***ANSWER:*** *The authors intend to analyze the references of the included articles to identify additional articles for inclusion in the review. This information has been clarified in lines 194-195.*

**RESPONSE TO REVIEWER #5.**

Reviewer #5 suggested the following rewordings:

**QUESTION 1) The description of materials and methods is thorough. The justification for why such an analysis is needed is well-described in the introduction. I would add at least a brief "Data Availability Statement" describing that all of your underlying data and analysis will be made available at submission to PLOS, and will be available publicly for review. You should consider adding to your introduction a little more language about how aspiration risk during cesarian section with local anesthesia is considerably low, and how often general anesthesia is utilized for C/S.**

***ANSWER:*** *To address the reviewers' comments, the "Data Availability Statement" section has been included (lines 269-272). Additionally, as guided by the current reviewer, more information about risk of aspiration has been added to the introduction (lines 61-65).*

**REFERENCES) Two new references have been added to address reviewer #5’s request, cited in lines 62 and 6, numbered 4 and 5 in the reference list. The PRISMA reference has been updated to the latest version, now numbered 11. These changes are highlighted in the reference list.**

**FINANCING) Study financing data was added in lines 2081 to 282.**
